# Systemic therapy for hereditary cancer

**DOI:** 10.1186/1897-4287-10-S4-A16

**Published:** 2012-12-10

**Authors:** Evgeny N Imyanitov

**Affiliations:** 1 N.N. Petrov Institute of Oncology, St.-Petersburg, 197758, Russia; 2 St.-Petersburg Pediatric Medical Academy, 194100, Russia; 3 I.I. Mechnikov North-Western Medical University, St.-Petersburg, 191015, Russia

## 

The history of specific therapy for hereditary tumors dates back to mid 1980s and involves a number of reports demonstrating regression of familial colon polyps upon administration of sulindac. Virtually no clinical studies on other hereditary cancer types were available until the year 2009, when Byrski et al. presented the data on unprecedented sensitivity of BRCA1-associated breast malignancies to cisplatin. This breakthrough has revived interest to the treatment of cancer in germ-line mutation carriers. Recent trials and clinical observations have confirmed the efficacy of platinating agents and PARP inhibitors in BRCA1/2-driven breast, ovarian and pancreatic carcinomas. Pegylated liposomal doxorubicin may be considered as a promising treatment option for BRCA1/2-related ovarian cancer after the failure of platinum-containing therapy. Several novel drugs have been recently introduced in the management of rare familial tumor syndromes. Vandetanib, a low-molecular weight RET kinase inhibitor, demonstrated substantial efficacy in the treatment of hereditary and sporadic medullary thyroid cancer. Vismodegib, an inhibitor of SMO oncoprotein, caused regression of basal-cell carcinomas in patients with Gorlin syndrome. Down-regulation of mTOR kinase by everolimus has been successfully used for the therapy of subependymal giant-cell astrocytomas in patients with tuberous sclerosis. The achievements in the prevention, diagnostics and treatment of hereditary cancers may serve as an excellent example of triumph of translational medicine.

